# Iatrogenic cerebral amyloid angiopathy and Alzheimer's disease co‐pathology

**DOI:** 10.1002/acn3.52278

**Published:** 2024-12-27

**Authors:** Francisco Hernández‐Fernández, Isabel Martínez‐Fernández, Rosa Barbella‐Aponte, Inmaculada Feria Vilar, Oscar Ayo‐Martín, Jorge García‐García, Rosa Collado, Alberto Andrés, Mar Hernández‐Guillamón, Francisco José Pena Pardo, Cristina Barrena, Miguel de la Fuente, Gemma Serrano‐Heras, María Melero, Elena Lozano Setién, Luis López, Tomás Segura

**Affiliations:** ^1^ Neurology Department Albacete Universitary Hospital Albacete Spain; ^2^ Pathology Department Albacete Universitary Hospital Albacete Spain; ^3^ Radiology Department Albacete Universitary Hospital Albacete Spain; ^4^ Research Unit Vall d'Hebron Universitary Hospital Barcelona Spain; ^5^ Nuclear Medicine Department Ciudad Real Universitary Hospital Ciudad Real Spain; ^6^ Neurosurgery Department Albacete Universitary Hospital Albacete Spain; ^7^ Research Unit Albacete Universitary Hospital Albacete Spain; ^8^ Internal Medicine Department Albacete Universitary Hospital Albacete Spain; ^9^ Neurology Department Vigo Universitary Hospital Vigo Spain; ^10^ Facultad de Medicina de Albacete Instituto de Biomedicina, UCLM Albacete Spain

## Abstract

Iatrogenic cerebral amyloid angiopathy, a disease caused by contact with neurosurgical material or human growth hormone contaminated by beta‐amyloid peptide (Aβ), has a prion‐like transmission mechanism. We present a series of three patients under 55 years of age who underwent cranial surgery. All of them developed multiple cerebral hemorrhages, transient focal neurological deficits, and/or cognitive impairment after 3–4 decades. MRI was compatible with CAA, and Aβ deposition was confirmed. The third patient, who had a ventriculoperitoneal valve, also showed Aβ deposition in the peritoneum and diagnostic biomarkers of Alzheimer's disease. Co‐pathology with Alzheimer disease and its iatrogenic transmission should be considered.

## Introduction

In recent years, a new form of cerebral amyloid angiopathy (CAA) named iatrogenic CAA (iCAA) has been identified. It affects young patients who underwent neurosurgical intervention 3–4 decades ago, in connection with the use of contaminated cadaver material such as dura mater grafts,^1^, ^2^ human growth hormone (HGH) injection,^3^ and/or surgical instruments.

iCAA is considered a disease with a prion‐like pathogenic mechanism. The prion‐like transmission mechanism has previously been suggested in Alzheimer's disease (AD) and other neurodegenerative diseases,^4^ and the ability of Aβ to propagate and induce misfolding of other proteins has been verified in mice and/or primates.^5^, ^6^

In 2015, iCAA was described in brain samples of patients with iatrogenic Creutzfledt‐Jakob disease (iCJD) who developed brain hemorrhages.^7^ Three years later, iCAA was experimentally demonstrated in mice by intracerebral inoculation of Aβ‐contaminated HGH preparations, leading to their deposition in cerebral vessels.^3^ Since then, several cases have been reported worldwide.^1^

Consequently, by 2022, diagnostic criteria had been established.^8^ In addition to a history of potential exposure, clinical and radiological manifestation characteristic of CAA need to be demonstrated, together with Aβ deposition in the CNS on amyloid PET, CSF, or brain biopsy, as well as the exclusion of hereditary causes of amyloidosis.

In addition to this new form of CAA, a recent paper^9^ has described the first cases of iatrogenic AD, demonstrating the Aβ deposition in parenchyma together with neurofibrillary tangles of P‐Tau.^9^ In the present report, we describe the characteristics of three patients who met criteria for iCAA seen in our hospital in recent years. One case also had clinical manifestations and CSF biomarkers diagnostic of AD.

The main aim of this study is to illustrate the clinical presentation and test findings in iCAA, and to improve the understanding of this emerging pathology.

## Methods

The diagnostic criteria applied were (1) age less than 55 years, (2) history of neurosurgical procedures (surgery on the brain, meninges, spinal cord, or posterior eye), embolization with freeze‐dried dura mater foam or administration of human hormones during childhood, (3) MRI that met the Boston 2.0 criteria for CAA,^10^ and (4) exclusion of other possible etiologies (see Appendix in Supplementary Material).

## Results

**Figure 1:**
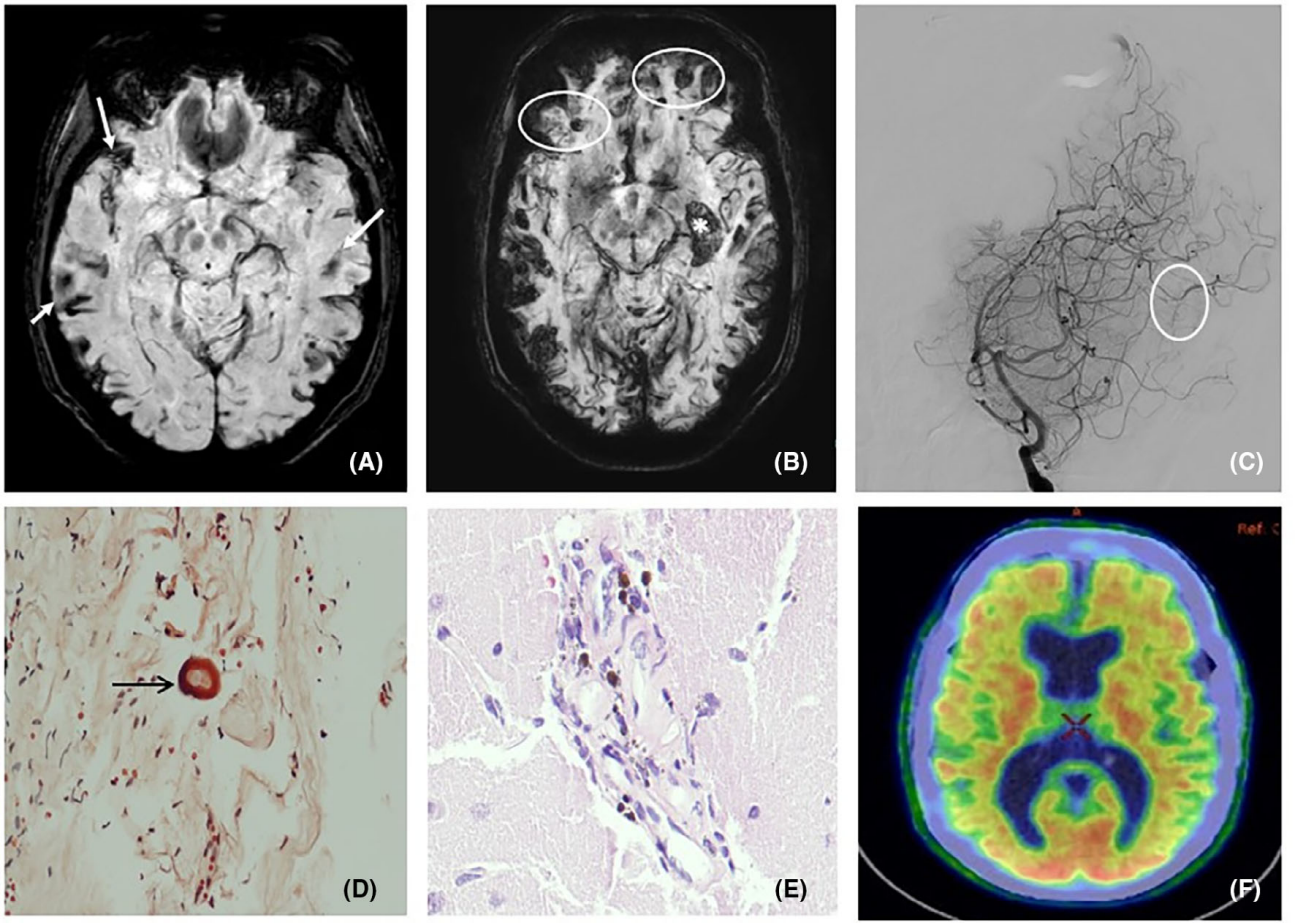
(A and B) axial MRI slices, in SWI sequence, from 2017 and 2023, respectively, which identify the existence of superficial siderosis (arrows), small subcortical microbleeds (circle), and intraparenchymal left temporal hematoma (*). Consistent with the diagnosis of probable cerebral amyloid angiopathy and significant progression between both studies. (C) Cerebral angiography showing moderate stenosis of the parietoocipital artery (circle). (D) Histological features of dura mater biopsy (Congo Red stain). Positive for amyloid deposition in the dura mater vessel (arrow). (E) Brain biopsy (hematoxylin‐eosin stain). The brain tissue shows reactive astrocytosis and neuropil edema. Vascular walls show hemosiderophages, lymphocytes, and plasma cells. (F) Axial section of 18F‐florbetaben PET‐CT scan. Isotope uptake in all the cortical reference areas.

**Figure 2:**
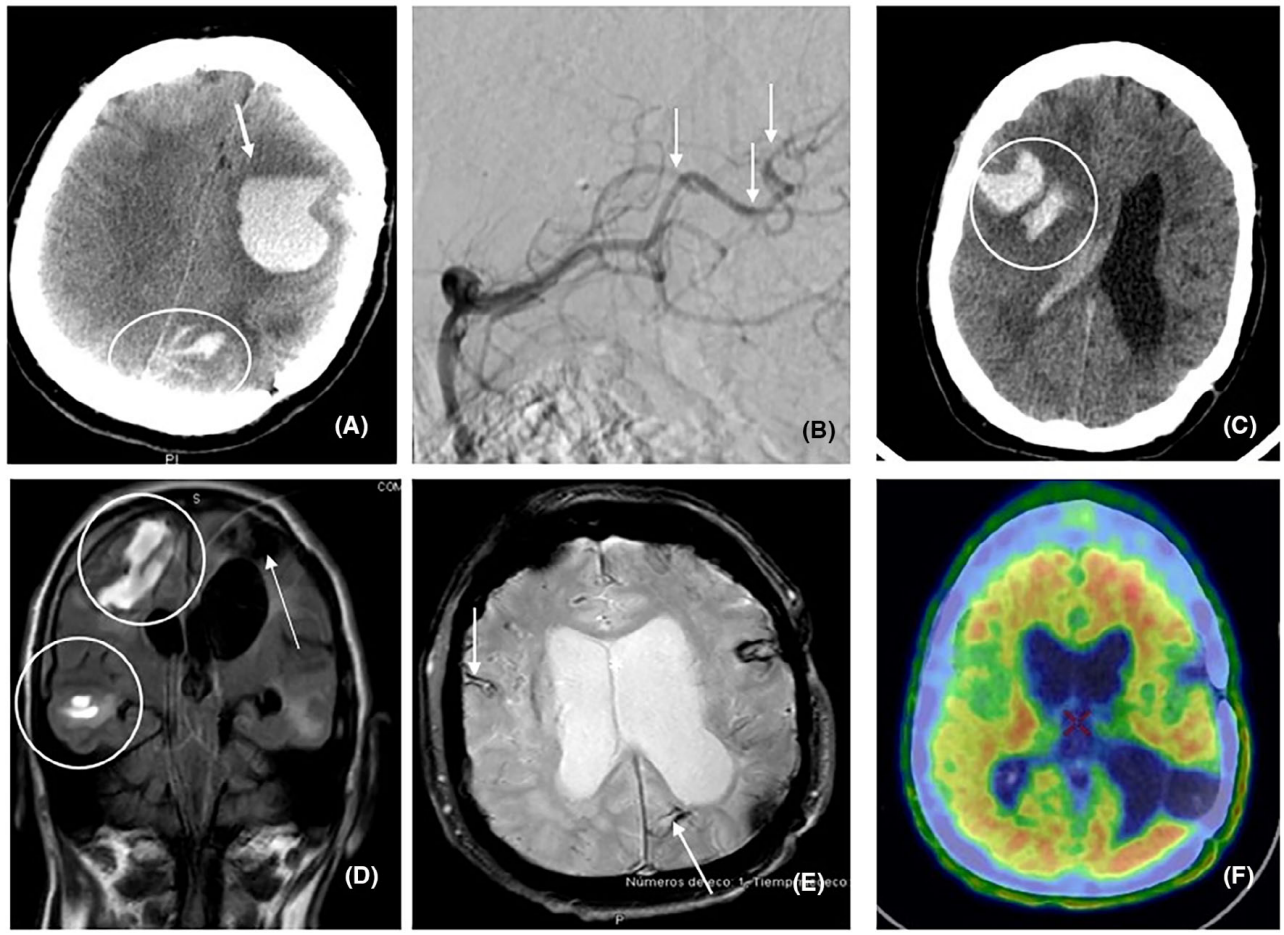
(A) Axial CT scan showing a left frontal hematoma (arrow), with surrounding edema. There is also a focus of SAH in the ipsilateral parietal convexity sulci (circle). (B) Cerebral angiography with selective run of the left vertebral artery, showing multiple changes and irregularities in the parito‐occipital branches of the posterior cerebral arteries (arrows). (C and D) Axial section of noncontrast CT (C) and coronal FLAIR MRI (D) showing an acute, heterogeneous, right frontotemporal hematoma (circles). The FLAIR image also shows sequelae of the previous hemorrhagic lesion (arrow), together with diffuse alteration of the white matter signal. (E) MRI, FFE T2* sequence showing sequelae of lobar intraparenchymal hemorrhages, together with cortical siderosis (arrows) and small microbleeds. (F) Axial section of 18F‐florbetaben PET‐CT scan. Isotope uptake in all the cortical reference areas.

**Figure 3:**
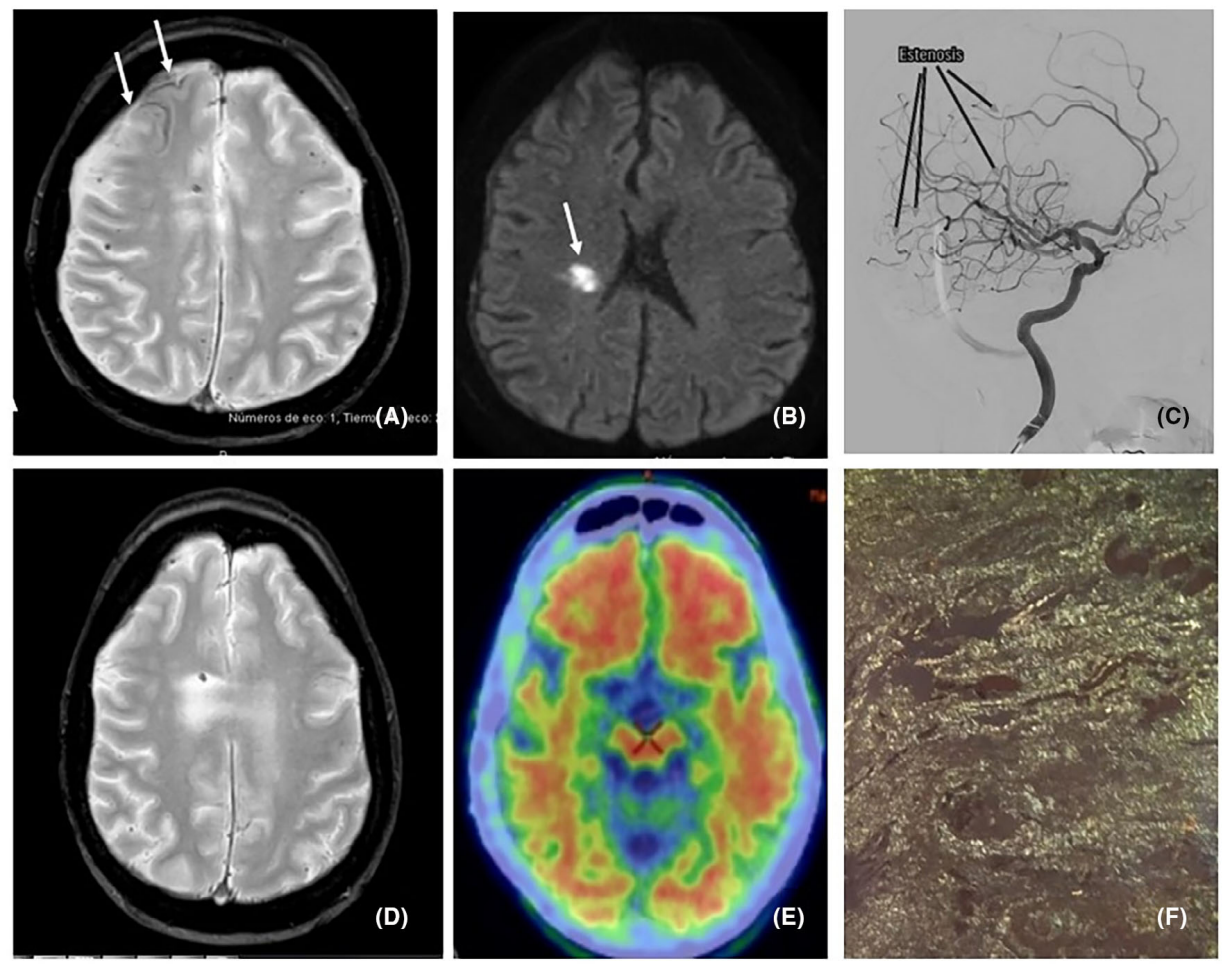
(A and D) axial MRI slices, T2*GRE, showing slight progression with the appearance of SAH in the right frontal convexity (Image A, arrows) and cortical microbleeds, not present in previous study (D). (B) axial DWI MRI sequence, showing an acute ischemic lesion in right corona radiata (arrow). (C) Cerebral angiography, showing multiple stenoses in medium and small arteries of the anterior circulation (arrows). (E) Axial section of 18F‐florbetaben PET‐CT scan. Cortical amyloid deposition in some reference areas, frontal and bilateral temporal. (F) Peritoneal biopsy. Congo red stain shows apple green birefringence under polarized light.

A total of three suspected cases were identified, as described in Table 1 and Figures 1, 2, 3. All of them underwent cranial surgery in childhood, and in patients 1 and 3, cadaveric dural grafts were used. The patients were younger than 55 years of age at the onset of symptoms. They presented with repeated transient focal neurological deficits, status epilepticus, and/or progressive mild cognitive impairment. Cranial MRI showed diffuse cortico‐subcortical or convexity subarachnoid hemorrhagic events, with progressive worsening. As shown in Table 1, Aβ deposition was confirmed by brain biopsy and/or 18F‐florbetaben amyloid PET. Cerebral angiography evidenced vasculitic changes, suggesting small vessel involvement. Given the early age of onset in the patients, genetic tests for CAA and familial amyloidosis were performed, as described in the Methods section, being negative for all of them.

**Table 1:** Patients with iCAA.

	Patient 1	Patient 2	Patient 3
Age at onset (year)	37 y.o. (2014)	49 y.o. (2016)	42 y.o. (2014)
Background information	Craneosynostosis surgery. CSF fistula repaired, Lyodura use (1978) Cocaine abuse	Left structural focal epilepsy Brain biopsy in childhood for seizures (1979), post‐TBI ischemia in cerebral tissue Second brain biopsy (2006), ganglioglioma in cerebral tissue No registered Lyodura use	Fourth ventricule subependymoma surgery (1978) Incomplete excision, local radiotherapy. Ventriculoperitoneal valve, several interventions due to malfunction Lyodura use.
Clinical evolution (and year)	Recurrent paroxysmal episodes of right sensory‐motor deficit and daily headache (2014) Headache and instability, paresthesias in right hemibody (2017, 2021 and 2023) No cognitive impairment nowadays (44 years‐old)	Left hemispheric status epilepticus (2016) Right hemispheric status epilepticus (2022)	Self‐limited motor aphasia and encephalopathy (2014) Prolonged paroxysmal episodes of intense agitation and headache, with or without aphasia and impaired consciousness. Progressive gait ataxia and cognitive impairment (2016–2022) Left hemiplegia (2024)
Image findings	Cortico‐subcortical microbleeds, SS, macrohemorrhages and cSAH (Fig. 1A) Extensive left temporal hemorrhage (2023) (Fig. 1B) Progressive worsening	Extensive left frontal hemorrhage (2016) (Fig. 2A) Right frontotemporal hematoma (2022) (Fig. 2C) New hemorrhage foci (2023) (Fig. 2D,E).	Right subcortical acute ischemic lesion, cortico‐subcortical microbleeds, SS, diffuse leukoencephalopathy progression (2024) (Fig. 3A,B)
CSF	Protein 0,54 g/dL, biomarkers not performed	Biomarkers in normal range (p‐tau 11.1 pg/mL, Aβ‐42/40 ratio 0.077), neurofilaments >10000 pg/mL	*Valve reservoir*: Protein 5 g/dL. Biomarkers compatible with Alzheimer's disease (p‐tau >400 pg/mL, Aβ‐42/40 ratio 0.042)
18F‐florbetabén amyloid PET	Positive for cortical amyloid deposition in all the cortical reference regions (Figs. 1F and 2F), and limited to frontal and temporal lobes (Fig. 3E)		
Cerebral angiography	Multiple nonspecific small vessel arterial stenoses (Figs. 1C, 2B, and 3C)		
Genetic testing for familiar CAA and amyloidosis	Negative		
Biopsy	*Cerebral biopsy (2023)*: Aβ deposition in dura mater and brain vessels. Moderate perivascular inflammatory component (Fig. 1D,E)	Not performed	*Peritoneal biopsy (2023)*: Aβ deposition (Fig. 3F)
Other interesting facts	Steroid and azathioprine treatment in 2021. Good response		Neuropsychological evaluation (2024): multi‐domain involvement with severe hippocampal dysfunction and impaired verbal episodic memory

cSAH, convexity subarachnoid hemorrhage; SS, superficial siderosis; TBI, traumatic brain injury.

It is important to highlight that the first patient also had a daily headache, poorly controlled with standard treatments. This symptom, together with the evidence of vasculitic changes, hyperproteinorrachia and cocaine abuse led us to suspect a drug‐induced inflammatory component. Immunosuppressive treatment was started (Table 1). Headache and paroxysmal episodes disappeared. Years after the cessation of drug consumption it was decided to discontinue corticosteroid treatment, but the symptoms reappeared and a new macrohemorrhage was evidenced on MRI. The treatment was reintroduced on suspicion of an inflammatory component associated with Aβ deposition, not in the context of cocaine use, and a biopsy was scheduled. Aβ deposition in dura mater and brain vessels was found (Fig. 1D), additionally highlighting a moderate perivascular inflammatory component (Fig. 1E). The fact that no cognitive impairment and no new events were observed, together with the stability of the MRI findings since 2023, support our inflammatory component hypothesis.

The third patient, who had a ventriculoperitoneal valve, was of special interest because of comorbidity with Alzheimer's disease. He presented with self‐limited motor aphasia some 36 years after surgery and local radiotherapy for ependymoma of the IV ventricle. Valvular normofunction was verified, and he was prescribed levetiracetam due to suspicion of an epileptic etiology. The patient experienced several prolonged paroxysmal episodes as described in Table 1, as well as progressive gait ataxia and cognitive impairment, but several electroencephalograms demonstrated encephalopathy with absence of epileptic activity.

Because of a sudden left extremity hemiplegia, an MRI was performed with data suggesting a right subcortical acute ischemic lesion (Fig. 3B) and cortical hemorrhage (Fig. 3A). The rest of the complementary tests were similar to the other two patients. A valvular malfunction intervention was performed due to obstructive hydrocephalus, obtaining a CSF sample from the ventricle. It revealed Aβ accumulation accompanied by an AD‐related pattern, as described in the Table 1: high levels of tau and P‐tau and low Aβ‐42/40 ratio. During revision of the distal catheter, a peritoneal biopsy was performed. This tissue was positive to Congo Red stain, but negative for AA‐amyloid immunohistochemistry (Fig. 3F). Currently, the patient presents a favorable physical recovery but moderate to severe cognitive impairment, especially with respect to bilateral hippocampal dysfunction and verbal episodic memory. On neuropsychological evaluation he presented a severe impairment in short‐term verbal learning, without benefit of semantic clues and inability to evoke elements in the long term. The impairment is moderate in visual episodic memory and also does not improve with item recognition. There is also mild impairment in semantic and phonological fluency, working memory, processing speed and premotor praxias, as well as orientation and attention.

## Discussion

Iatrogenic transmission of Aβ mediated by a prion‐like mechanism was described in 2015 and experimentally confirmed in 2018.^3^, ^7^ The criteria proposed in 2022 by Banerjee *et al*.^8^ facilitate the search of new cases. A history of potential exposure to a relevant neurosurgical procedure is necessary, even more when human cadaveric CNS (HGH or Lyodura) tissue is used. In addition to the clinical and radiological manifestations indicative of probable CAA, it is necessary to demonstrate Aβ deposition in the CNS with amyloid PET, CSF biomarkers, or brain biopsy, together with the exclusion of hereditary causes of amyloidosis. Suspicion of probable iCAA will be higher in patients with clinical onset at ages below 55 years, as it is a rare disease below the age of 30 years due to its very long latency period.^1^ However, it should not be ruled out in patients over 55.

Other mechanisms may be involved in the excessive accumulation of Aβ in the CNS, such as the innate and humoral immune response.^11^, ^12^ In our first case, the brain biopsy showed glial activation and moderate perivascular inflammatory exudate. The symptomatic improvement after immunosuppressive treatment may support this idea.

Another mechanism that may facilitate amyloid deposition is impaired drainage by the glymphatic system. It enhances Aβ and other waste particles removal, by allowing solutes to move through the parenchyma via astroglial water transport (AQP4) from the arteriole to the venules.^13^ Cadaver dural grafts could potentially interfere with the natural drainage mechanisms. Despite the poor understanding of the anatomical and physiological relationship between the brain parenchyma and the subarachnoid space, it is known that CSF stagnation favors vascular and parenchymal deposition of Aβ and tau.^14^ It is interesting to mention a pilot study in AD with a low‐flow shunt placed with the aim of increasing CSF circulation and turnover.^15^ Indeed, the mere placement of a ventricular catheter, regardless of whether an active shunt is present, can generate a CSF pathway around the periphery of the catheter. Although the confirmatory study was terminated for futility,^16^ the possible implications on prodromal AD or vascular deposition of Aβ remain unknown.

This could have interesting implications for the development of potential therapeutic targets that could accelerate Aβ clearance. In particular, the peritoneal biopsy findings in our third case open up the possibility of extracerebral clearance of Aβ via CSF shunting. The relationship between Aβ deposition in the CNS and peritoneum has already been explored in patients with normal pressure hydrocephalus^17^ and in murine models that demonstrated the ability to induce cerebral beta‐amyloidosis when Aβ was inoculated peripherally into the peritoneum.^6^

In this context, it is worth noting the striking clinical and radiological differences between our patients, which may provide valuable data. Our third patient, with a ventriculoperitoneal valve since childhood had a less aggressive clinical and radiological presentation and evolution. He presented with major neurocognitive symptoms meeting AD criteria along with recurrent encephalopathy and relatively minor hemorrhagic involvement. We hypothesize that this may be related to the clearance of Aβ to the peritoneum through the valvular shunt. By increasing drainage from perivascular CSF (and, consequently, from the vascular system), less CSF vessel deposition could be favored.

The clearance that reduces Aβ deposition in the vessels may not be extrapolate to the parenchyma. In this sense, the finding of reduced Aβ‐42/Aβ‐40 ratio and high P‐tau in the CSF levels in this case are noteworthy. This deposition pattern is the biological hallmark of AD and the least frequently identified in iCAA registries.^18^ According to the latest reviews, it is not present in other amyloidopathies.^19^ Regarding the possibility of iatrogenic AD, a series of five patients following HGH treatment has been recently described.^9^ In addition, three patients diagnosed with iCAA whose brain biopsy met diagnostic criteria for AD have also been identified in the United Kingdom.^20^ All of them, like our patient, share the significant increase of p‐tau in CSF. They also share an atypical expression of AD, with multi‐domain involvement at an early age and presence of additional neurological signs, such as progressive ataxia or episodes of encephalopathy. In line with these case series, our patient number 3 lacks prominent hemorrhagic manifestations and has limited evidence of CAA on amyloid PET. These findings suggest iCAA and co‐pathology with AD are probably the same entity with a different clinical expression.

In conclusion, iCAA is a new emerging disease of unknown prevalence and prion‐like mechanism of transmission after neurosurgical procedures. We report a series of three new cases, one of which meets diagnostic criteria for comorbidity with AD. He has a similar clinical profile to the cases described by Banerjee *et al*.^9^, ^20^ and is the first case diagnosed anywhere outside the United Kingdom, which blurs the possible geographical limits of this pathology. In addition, it provides new data which could contribute to improving the understanding of this disease, as well as other neurodegenerative diseases with this type of transmission.

## AUTHOR CONTRIBUTIONS

F. H.‐F. and I. M. F.: Conception and design of the study; acquisition and analysis of data; drafting a significant portion of the manuscript or figures; approval of the final version. R. B.‐A., O. A.‐M., J. G. G., A. A., M. H.‐G., and T. S.: Acquisition and analysis of data; drafting a significant portion of the manuscript or figures; approval of the final version. I. F. V., R. C., F. J. P. P., C. B., M. D. F., G. S.‐H., M. M., E. L. S., and L. L.: Acquisition and analysis of data; approval of the final version.

## Funding Information

There is no funding sources related with this paper.

## Conflict of Interest

There is no conflict of interest related with this paper.

## Supporting information


Data S1.

## Data Availability

The raw data supporting the conclusions of this article will be made available by the authors, without undue reservation. Data analysis was made by FHF, IMF, and IFV.
