# Supplementary material for: Iatrogenic cerebral amyloid angiopathy and Alzheimer's disease co‐pathology
Source: Ann Clin Transl Neurol. 2024 Dec 27;12(1):235–41. doi: 10.1002/acn3.52278 (PMC11752100; doi:10.1002/acn3.52278)
Supplement: Supplementary file 1 — Data S1. [file ACN3-12-235-s001.docx]

**APPENDIX**

In our centre, a protocolised study is performed in cases of cerebral haemorrhage in patients under 56 years of age, which includes: cerebral arteriography, echocardiography, blood tests (haemogram, biochemistry, coagulation, autoimmunity, serology for sexually transmitted infections, borreliosis, cytomegalovirus, brucellosis, hepatotropic viruses) and CSF analysis (biochemistry, culture, serology). Those cases with deep bleeding and no history of arterial hypertension, the study was completed with a genetic panel for microangiopathy. In those with positive Boston Criteria for CAA, a genetic study of familial amyloidosis was performed with massive exome sequencing in blood (APOA1-2, APOC2, APP, ATP13A2, B2M, CHCHD10, CHMP2B, CSF1R, DNAJC5, DNMT1, EPM2A, FGA, GRN, GSN, ITM2B, LYZ, NHLRC1, NLRP3, NOTCH3, PLA2G6, PRNP, PSEN1-2, SNCA, SNCB, SQSTM1, TARDBP, TBK1, TIMM8A, TREM2, TTR, TYROBP, UBQLN2, VCP, XK). The CSF determinations (Ab42, Ab40, t-Tau, p-Tau181) were carried out by chemiluminescence immunoassay (Lumipulse, Fujirebio, Tokyo, Japan) in the clinical laboratory from the University Hospital of Albacete. The reference levels are: Tau [146-410 pg/mL], P-Tau [21.5-59 pg/mL], βA-42 [725-1777 pg/mL], βA-40 [7755-16715 pg/mL], βA-42/40 ratio [0.068-0.115]. When the sample collected was sufficient, the level of Neurofilament Light Chain (NFL) was quantitatively determined. In patients with cognitive impairment, neuropsychological tests used were: TBA-2 (abbreviated Barcelona test), FCSRT (free and cued selective reminding test), and cancellation test.

Tissues biopsies were stored in 4% formaldehyde, then embedded in paraffin, microtomed into 4-μ sections, and prepared using the established histological protocol, with hematoxylin–eosin and Congo red stain. Immunohistochemistry in nervous and non-nervous tissues was performed using monoclonal antibodies against amyloid fibril protein AA (Monoclonal mouse Anti-Human Amyloid A, Clon mc1, Ready-to-Use, Agilent).
